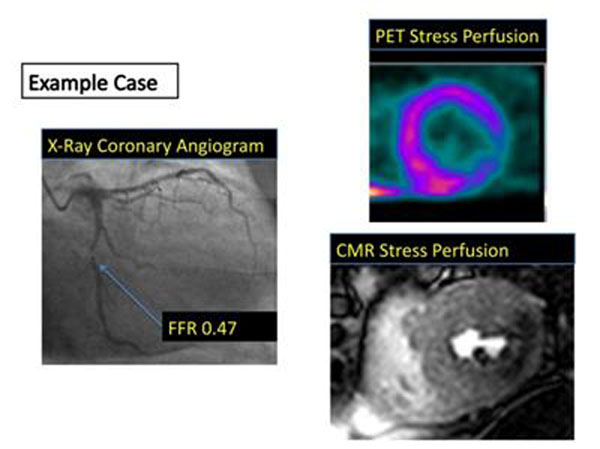# Comparison of cardiac magnetic resonance imaging and positron emission tomography for the diagnosis and localization of coronary artery disease

**DOI:** 10.1186/1532-429X-13-S1-P83

**Published:** 2011-02-02

**Authors:** Geraint Morton, Masaki Ishida, Amedeo Chiribiri, Andreas Schuster, Stacey Baker, Shazia Hussain, Divaka Perera, Michael O'Doherty, Sally Barrington, Eike Nagel

**Affiliations:** 1 King's College London, London, UK

## Objective

Compare the diagnostic performance of Cardiac Magnetic Resonance (CMR) myocardial perfusion imaging against Positron Emission Tomography (PET) in coronary artery disease (CAD).

## Background

PET is regarded as the non-invasive reference-standard for assessment of myocardial perfusion. However, there are few data comparing the performance of CMR against PET perfusion imaging, particularly in patients with a high prevalence of CAD. In addition, novel MR techniques, e.g. based on kt acceleration techniques, allow perfusion imaging with improved spatial resolution.

## Methods

22 patients with known or suspected CAD underwent both ^13^N-Ammonia PET and CMR adenosine stress and rest perfusion imaging prior to diagnostic coronary X-ray angiography (CXA). CMR perfusion imaging was performed at 1.5T with a kt-accelerated steady-state free-precession sequence.

Data analysis was blind. A significant coronary artery stenosis was defined as at least 70% diameter reduction or a fractional flow reserve <0.8. Sensitivity and specificity for PET and CMR versus invasive angiography were calculated. Localization of ischaemia was assessed in patients with CAD by classifying territories that were ischaemic on imaging as either supplied by, or as remote from, a stenotic artery.

## Results

Patient characteristics-table 1. 1 CMR examination was non-diagnostic. The prevalence of CAD was 82%. Mean interval ± standard deviation between PET and CMR scans was 3±7 days (73% same day); between PET and CXA was 19±21 days and between CMR and CXA 20±23 days.

**Table 1:** Patient characteristics

Age (Mean±SD)	64±10 years
Male	77%
Diabetes	32%
Previous percutaneous coronary intervention	32%
Hypertension	59%

For the detection of CAD PET sensitivity was 83% (95% CI 58-96%) and specificity 75% (95% CI 22-99%). CMR sensitivity was 82% (95% CI 56-95%) and specificity 75% (95% CI 22-99%). In the 18 patients with CAD there was detectable ischaemia in 79% of the coronary artery territories supplied by significantly stenotic arteries with both PET and CMR. Ischaemia was also detected in 20% of remote territories with PET and 17% with CMR. 50% (n=3) of the territories with remote ischaemia on PET imaging also had remote ischaemia on CMR imaging.

## Conclusions

Perfusion CMR imaging appears to be as good as PET for the diagnosis of CAD. However the numbers are relatively small so confidence intervals are wide and further work is required. In patients with CAD localization of ischaemia to a coronary territory is comparable with both modalities. Using an anatomic test as the reference-standard for functional tests has well-described limitations. Remote ischaemia is likely to occur for several reasons including underestimation of disease severity at CXA, microvascular disease and also false positive results.

**Figure 1:**